# A Novel Approach to Better Characterize Medication Adherence in Oral Anticancer Treatments

**DOI:** 10.3389/fphar.2018.01567

**Published:** 2019-01-29

**Authors:** Marie Paule Schneider, Leila Achtari Jeanneret, Bernard Chevaux, Claudine Backes, Anna Dorothea Wagner, Olivier Bugnon, François Luthi, Isabella Locatelli

**Affiliations:** ^1^Community Pharmacy, School of Pharmaceutical Sciences, University of Geneva, University of Lausanne, Geneva, Switzerland; ^2^Community pharmacy, Department of Ambulatory Care and Primary Medicine, University of Lausanne, Lausanne, Switzerland; ^3^Hôpital Neuchâtelois, Neuchâtel, Switzerland; ^4^Etablissements Hospitaliers du Nord Vaudois (eHnv), Yverdon-les-Bains, Switzerland; ^5^Department of Oncology, Lausanne University Hospital (CHUV), Lausanne, Switzerland; ^6^Hirslanden SA Lausanne, Clinique Bois-Cerf, Lausanne, Switzerland; ^7^Department of Ambulatory Care and Primary Medicine, University of Lausanne, Lausanne, Switzerland

**Keywords:** medication adherence (MeSH), electronic monitoring (EM), oral treatments, oncology, non-persistence, routine care, censoring, generalized estimating equations (GEE)

## Abstract

**Purpose:** This study aims to describe a 12-month medication adherence with oral anticancer medications (OAMs) in a routine care medication adherence program, and to better characterize non-persistence.

**Patients and methods:**In this observational, one-centered, longitudinal study, medication adherence was monitored electronically while patients were taking part in a medication adherence program for 12 months or until treatment stop. Patients were >18 years and starting or taking one of the following OAMs: letrozole, exemestane, imatinib, sunitinib, capecitabine, or temozolomide. Non-persistence was defined as any premature treatment interruption due to patient's unilateral decision or to a medical decision because of adverse effects. The Kaplan Meier survival function estimate was used to characterize persistence, and Generalized Estimating Equations (GEE) were adopted to fit implementation. Statistical analyses were performed using the R software package.

**Results:** Forty-three outpatients with various tumor entities were enrolled. Reasons for quitting the medication adherence program and/or OAM medication were characterized as OAM discontinuation due to adverse effects or toxicity (*n* = 5), planned OAM completion time (*n* = 10), OAM failure (cancer relapse) (*n* = 5) and non-compliance to the adherence program (*n* = 3). In persistent patients, the implementation rates were high (from 98% at baseline to 97% at 12 months). The probability of being persistent at 12 months was estimated at 85%.

**Conclusion:** A better characterization of both persistence and implementation to OAMs in real life settings is crucial for understanding and optimizing medication adherence to OAMs. The complex identification of non-persistence underlines the need to carefully and prospectively assess OAM interruption or treatment switch reasons. The GEE analysis for describing implementation to OAMs will allow researchers and professionals to take advantage of the richness of longitudinal real-time data, to avoid reducing such data through thresholds and to put them into perspective with OAM blood levels.

## Introduction

Cancer treatments used to be administered solely by intravenous (i.v.) infusions during patients' hospital visits. The increasing availability and use of oral anticancer medications (OAMs) has shifted patient therapy schemes from outpatient oncology units to home-administered settings.

Anticancer therapy investigations are making outstanding approaches available to enhance efficiency by focusing, for example, on targets. As a result of scientific progress, cancer patients' life expectancies have improved substantially, and changed the disease and thus the medications from acute to chronic treatments, e.g., in breast cancer, leukemia, gastrointestinal stromal tumor (GIST). Chronic cancer patients face major new challenges, such as the optimization of long-term adherence to cancer medication under orally administrated regimens (Sabate, 2003; Ruddy et al., 2009; Weingart et al., 2010; Wood, 2012; Timmers et al., 2014). However, data on adherence to OAMs in real life settings remain scarce.

Compared to inpatient intravenous (i.v.) administration, OAMs are associated with improved quality of life for patients, as they eliminate the need for invasive drug administration, enhancing patients' autonomy, and responsibility and allowing home-treatment (Ruddy et al., 2009). Nonetheless, the narrow therapeutic index of OAMs requires close monitoring of adverse effects to ensure treatment safety, and a high medication adherence to ensure treatment efficacy.

Medication adherence is considered as an overall definition including treatment initiation, implementation and persistence (Vrijens et al., 2012). The implementation characterizes the day-by-day drug intake during the period of persistence. Persistence is defined as the time period from treatment initiation until discontinuation. Even if no “gold standard” measurement method exists, electronic monitoring (EM) represents the most accurate method to capture the dynamic and longitudinal characteristics of drug intake (Krummenacher et al., 2010; Lehmann et al., 2014; Nguyen et al., 2014).

Published OAM adherence rates were reported to vary largely, i.e., from 16 to 100%, and included mainly clinical trials data (Partridge et al., 2002, 2003; Bhattacharya et al., 2012; Allen and Williamson, 2014; Barillet et al., 2015). Rates differed according to cancer type and stage, and according to used adherence measurement methods. Determinants associated with non-implementation and non-persistence in cancer patients are complex and interrelated (Cheung et al., 2015; Verbrugghe et al., 2016). In breast cancer, for example, non-persistence is predominantly caused by treatment-related adverse effects, longer-term therapies and age (Verbrugghe et al., 2016). Sub-optimal implementation and persistence rates of long-term OAMs in routine care are particularly problematic, as the border between medication toxicity and cancer progression due to inadequate plasma concentrations is not well established. Additionally, considering the constantly increasing number of prescribed targeted OAMs, non-adherence needs to be better characterized and knowledge needs to be transferred into practice in order to support patient-tailored and interprofessional adherence interventions in oncology (Hershman et al., 2011; Ibrahim et al., 2011; Al-Barrak and Cheung, 2013).

The pharmacy of the Department of Ambulatory Care & Community Medicine in Lausanne (PMU) runs an interprofessional medication adherence program (IMAP) (Lelubre et al., 2015). Medication adherence to each drug is monitored via electronic monitoring, combined with manual pill count and feedback to the patients. This study aims to describe a 12-month adherence -persistence and implementation- to different OAMs in a routine care medication adherence program, and intends to better characterize discontinuation and non-persistence by analyzing reasons for discontinuation.

## Methods

This is a one-center, observational, longitudinal study approved by the Ethics Committee of Canton de Vaud, Switzerland (Protocol Nr: 261/07). Informed and written consent was obtained from all study participants. Inclusion criteria were: patients older than 18 years, starting or taking an on-going OAM, ability to use electronic monitoring (EM) devices, and absence of severe psychiatric or social disorders, or language barrier.

Following OAMs were included with their respective intake schemes: letrozole (aromatase inhibitor) 2.5 mg QD on a continuous scheme; exemestane (aromatase inhibitor) 25 mg QD on a continuous scheme; imatinib (tyrosine kinase inhibitor) 400, 600, or 800 mg a day, QD or BID on a continuous scheme; sunitinib (tyrosine kinase inhibitor) 37.5 mg QD on a continuous scheme or 50 mg QD 4 weeks on/2 weeks off; temozolomide (alkylating agent) 75 mg/m^2^/day concomitantly to radiotherapy or 250 mg/m^2^/day, 5 days on/3 weeks off; or capecitabine (5-fluorouracil prodrug) 2,000–2,500 mg/m^2^/day BID 2 weeks on/1 week off. Neither a dose change in regimen, nor a switch between OAMs during the 12-month study was a criterion for participant's withdrawal.

The exploratory analysis was based on the enrolment of a convenient sample of approximately 40 consecutive participants. Participants were monitored for 12 months or until treatment stop with participants' study visits scheduled at inclusion, at 3, 6, 9, and 12 months.

Medication adherence was monitored electronically. All participants used EM with an electronic display on top (MEMS SmartCapTM, Medication Event Monitoring System, AARDEX Group, Sion, Switzerland; Krummenacher et al., 2010; Lehmann et al., 2014; Lelubre et al., 2015). EM consists of an electronic device that records and stores the date and time of each pill container opening. The electronic display on top of EM informs the patient in real time on the number of daily pill-bottle openings (e.g., 0, 1, 2, etc.). At each study visit, EM results were printed in a report format, which summarizes graphically the drug intake since the last study visit, and were discussed with each participant (Lelubre et al., 2015). To increase reliability, electronic data were reconciled according to an operational manual previously described (Rotzinger et al., 2016). Reconciliation with pill count and interview notes allowed for the addition of confirmed pocket doses to the electronic adherence data, i.e., a dose that the patient removes from the pillbox to swallow later.

All consecutive patients were enrolled by the hospital oncologists whatever their level of adherence and referred to the pharmacists of the Department of Ambulatory Care & Community Medicine in Lausanne (PMU) (Lelubre et al., 2015). Each prescribed OAM was dispensed in a separate EM device according to good pharmaceutical practices. At inclusion, the correct use of the EM device was explained and opportunity to practice was given. Missed study visits at the adherence clinic were rescheduled.

Participants' socio-demographic characteristics, clinical and treatment data were collected at baseline by reviewing the medical and pharmacy records.

The ABC European adherence initiative taxonomy of persistence, implementation and adherence was used in this study (Vrijens et al., 2012) as described below:

1. “Persistence” was defined as the distribution of times until OAM discontinuation. OAM discontinuation was defined here as a medication stop due to either the patient own, unilateral decision or a medical decision because of an OAM adverse effect or toxicity. Stops due to OAM failure or planned end of treatment (completion, cancer remission, surgery, or prescribed switch to other iv oncology or radiology therapies), due to non-compliance to the medication adherence program without medication discontinuation (participants quitting the medication adherence study), as well as the end of the 1 year observation period were considered as *censoring*.
2. “Implementation” at day *x* was expressed as the number of participants who took at least the prescribed medication dosing regimen at day *x* divided by the number of participants not having discontinued, nor being censored before that day (Blaschke et al., 2012).
3. “Adherence” at day *x* represented the number of participants who took at least the prescribed medication dosing regimen at day *x* divided by the number of participants initially included into the study.

### Data Analysis

Classical descriptive statistics were used to describe socio-demographic data at baseline, and clinical data at baseline and at exit. Numbers and percentages were adopted to characterize binary and categorical variables; median and 1st and 3rd quartiles were used to summarize quantitative variables.

Implementation was plotted against time and compared with the number of participants still under observation (neither discontinuing, nor censored) at each day x. Persistence was estimated using the Kaplan Meier survival function of discontinuation times.

Censored durations before the end of the observation period represented a challenge in calculating adherence. By definition, a censored participant is a participant who will experience discontinuation somewhere in the future, without information on the exact time. Thus, a censored patient will certainly continue taking medication for a period after the censoring date, but daily adherence during this period is unknown since patient is no more under observation. In addition, the possibility of a discontinuation before the end of the study, with a subsequent null adherence, cannot be excluded. A somehow natural solution could consist in considering adherence after censoring as missing (*naive adherence curve*). Unfortunately, the resulting adherence would be inconsistent with the Kaplan Meier estimate of persistence. The latter in fact jumps only when a discontinuation occurs, the size of each jump depending on the number of censoring having occurred before each discontinuation (Zhang et al., 2009), while the naive adherence curve drops at discontinuations *and* (to a lower extent) at censoring times, leading to an estimate of adherence at the end of follow up systematically lower than the Kaplan Meier estimate of persistence. Persistence representing at a given day the proportion of participants not having discontinued at that day, adherence must coincide with this proportion if implementation is perfect (= 100%) on that day (adherence = persistence). Since this is not true for the naive adherence curve (adherence < persistence), we conclude that the latter represents a *biased* estimate of adherence.

Therefore, the presence of censoring prevents us from calculating adherence *directly* from censored data. We propose here to estimate adherence *indirectly* as the product, for each follow-up time, between implementation and the Kaplan Meier estimate of persistence (i.e., implementation^*^persistence). Such a solution, which gives the same results as the empirical adherence in an ideal situation where there is no censoring, was considered as the optimal one in our setting, where adherence cannot be calculated directly on data because of censoring.

Generalized Estimating Equations (GEE) with an “independence” correlation structure were adopted in order to fit implementation. Time was entered into the model using “splines” (two knots at 4 and 8 months), allowing a flexible estimation of the implementation pattern across time. Confidence intervals around implementation were obtained using a robust estimation of the covariance matrix of the model parameters.

Statistical analyses were performed using the R software package (R Core Team, 2013).

## Results

A total of 51 patients were eligible. Eight patients refused to participate in the study; six were not interested, one individual considered the study as too inconvenient and one elderly individual did not feel at ease with EM handling. Thus, the study included 43 participants.

The study lasted from March 2008 to October 2011, and provided 12'081 cumulated days of observations (openings of EM device). Participants' socio-demographic data at baseline, and clinical data at baseline and exit are summarized in Table 1. The majority of participants [median age 62 (52–69), 53% women] was naive to treatment (88%) and was diagnosed with gastrointestinal stromal tumor or breast cancer. Median time length between cancer diagnosis and study initiation was seven months.

**Table 1:** Participants socio-demographic data at baseline, and clinical data at baseline and at exit.

**SOCIO-DEMOGRAPHIC DATA (*N* = 43)**			
Age (in years) median [Quartiles 25 and 75]		62	[52, 69]
Gender	Women	23	53%
	Men	20	47%
**CLINICAL DATA AT BASELINE**			
Cancer types	Gastrointestinal Stromal tumor	21	49%
	Breast cancer	17	40%
	Kidney cancer	4	9%
	Brain cancer	1	2%
Duration (months) between diagnosis and start of the study [median, IQR]		7	[2,27]
Study start within 2 months of treatment initiation	Yes	31	72%
	No	12	28%
**MONITORED ORAL ANTICANCER TREATMENT (OAM)**			
Name of OAM^1^ (DCI)	Capecitabine	10	24%
	Letrozole	10	24%
	Imatinib	9	20%
	Sunitinib	7	16%
	Exemestane	6	14%
	Temozolomide	1	2%
Prescribed pharmacological regimen	Once a day (QD)	33	76%
	Twice a day (BID)	10	24%
Prescribed daily dose for each drug treatment Median ± (IQ)	Capecitabine	2.8 g	[2.0; 5.0]
	Letrozole	2.5 g	[2.5; 2.5]
	Imatinib	400 mg	[400; 400]
	Sunitinib	37.5 mg	[12.5; 56.25]
	Exemestane	25 mg	[25; 25]
	Temozolomide	130 mg	[130; 130]
Treatment scheme	Continuous	37	86%
	Cyclic	6	14%
Treatment line	1st treatment (naive patients)	38	88%
	2nd treatment	3	7%
	≥3rd treatment	2	5%
Purpose of the treatment	Adjuvant	25	59%
	Palliative	12	29%
	Neo-adjuvant	5	12%
Type of concomitant cancer treatments	Oral medication	1	2%
	Intravenous therapy	8	20%
	Radiotherapy	6	14%
	None	27	64%
**CLINICAL DATA AT THE END OF THE STUDY (AT 12 MONTHS OR UPON WITHDRAWAL FROM THE STUDY)**			
Disease prognosis	Remission	26	60%
	Partial tumor response	13	31%
	Cancer progression	4	9%

1 *OAM, oral anticancer medication*.

**Figure 1:**
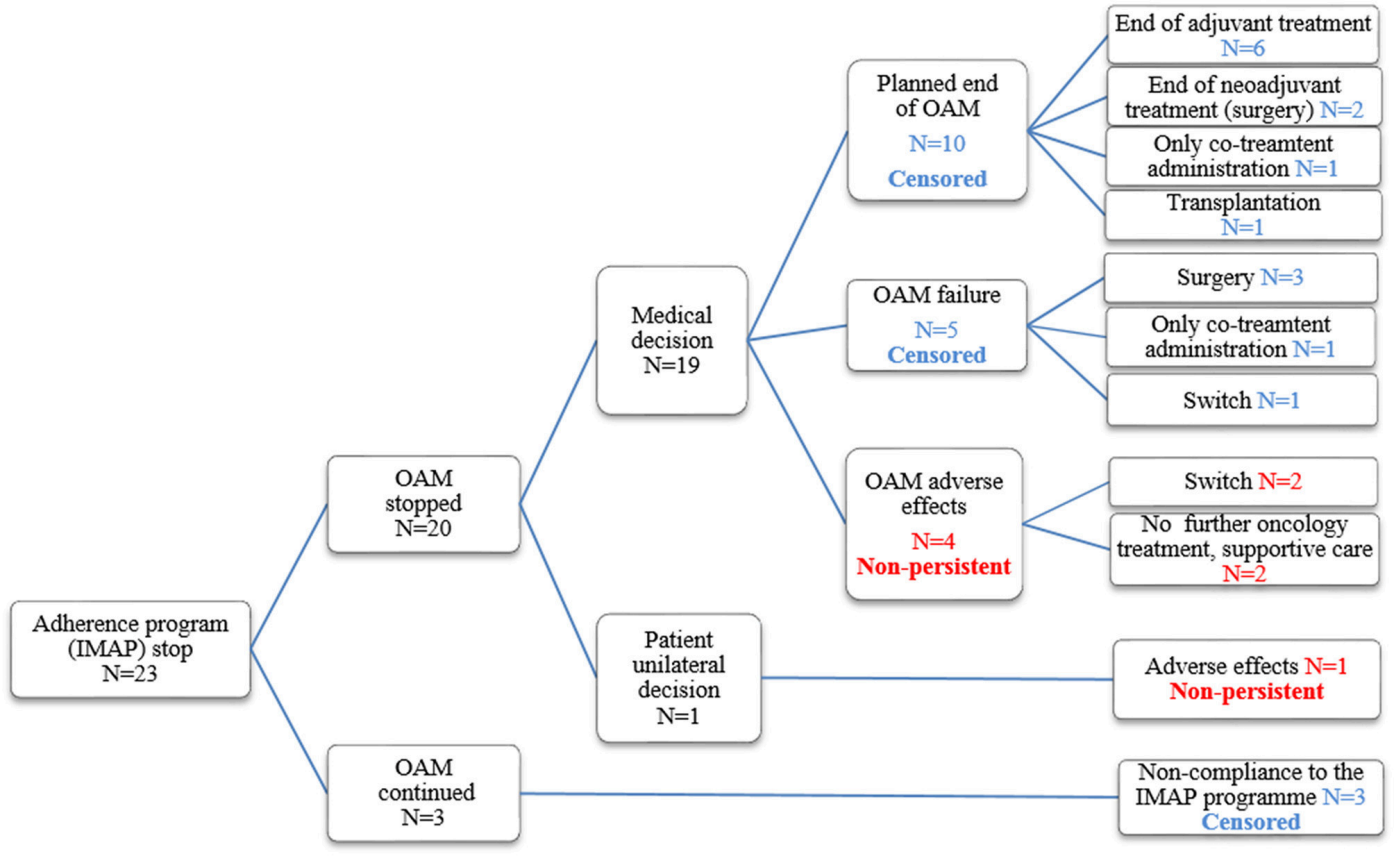
Reasons for medication adherence program stop and/or OAM stop.

The reasons for quitting the medication adherence program and/or OAM medication before the 12-month completion of the observation period are illustrated in Figure 1. Among the 23 participants (23/43 inclusions, 53%) having quitted the medication adherence program, 5 OAM discontinuation cases were identified, all due to adverse effects or toxicity. Of these five, one participant decided to discontinue his OAM unilaterally without any medical agreement and four participants discontinued their OAM with medical agreement. The array of adverse effects was quite large from acute renal failure, severe mental health problems, oedema, unstable angina, and insomnia. Eighteen participants having quitted the adherence program were censored due to following reasons: (i) 10 cases of OAM planned completion time, (ii) 5 cases of OAM failure, (iii) 3 cases of non-compliance to the IMAP medication adherence program but with OAM continuation.

**Figure 2:**
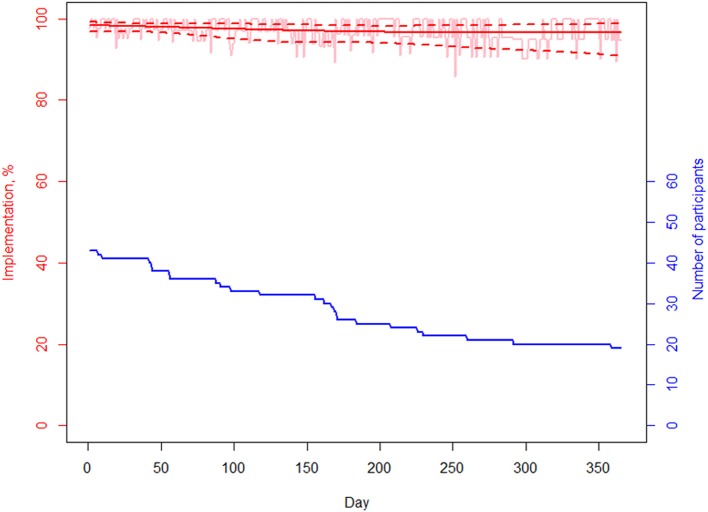
Implementation. Empirical curve and fit obtained with GEE model.

**Figure 3:**
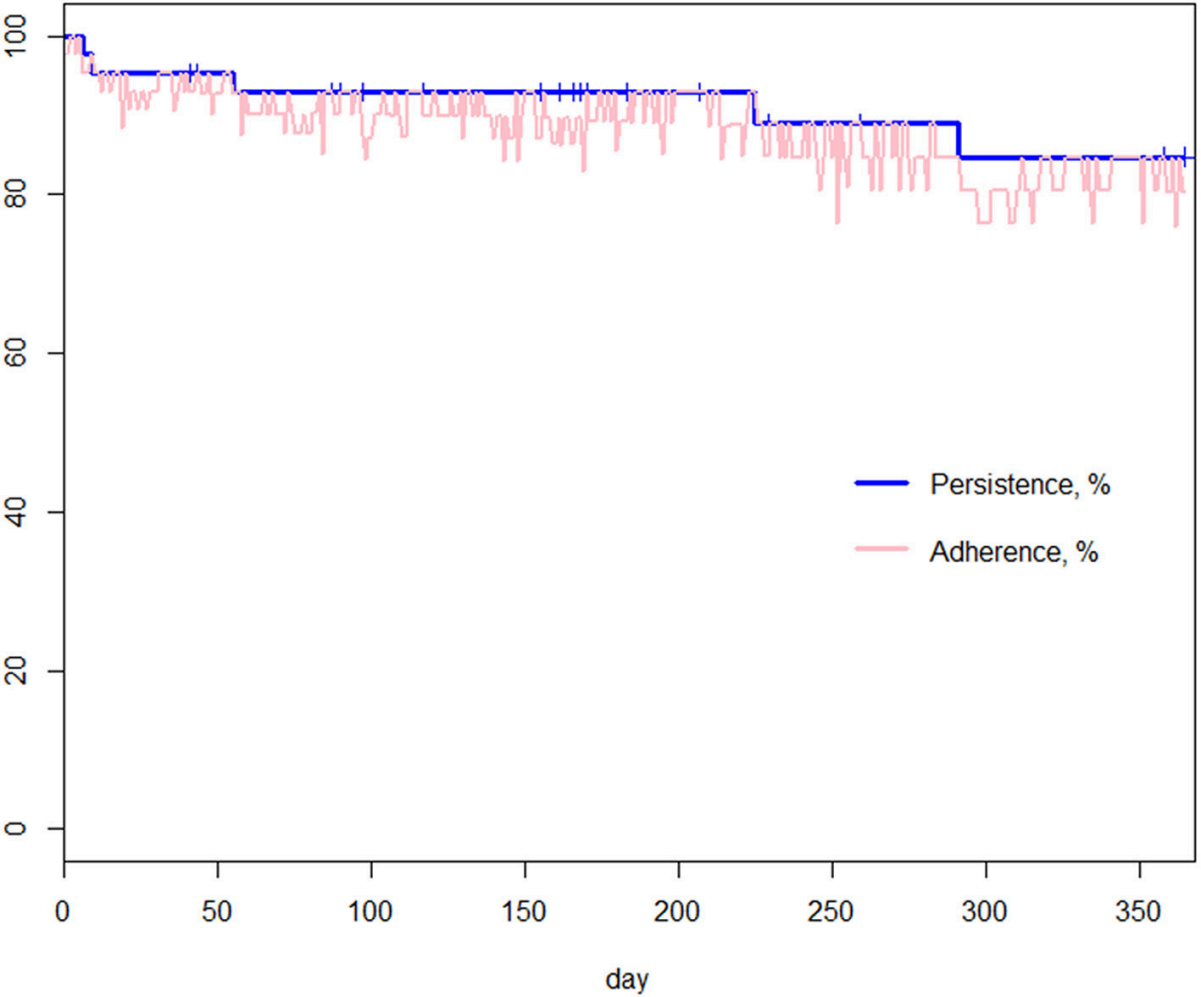
Adherence and persistence. Blue curve: Kaplan Meier estimator of persistence: it represents an estimate of the percentage of patients not having discontinued as a function of time. Pink curve: Adherence obtained multiplying implementation and persistence: it represents an estimate of the daily percentage of patients, among those initially included into the study, correctly taking the prescribed medication.

Figure 2 shows implementation over time. Implementation remained stable but with an increase in variability. The latter was due to the decrease of the number of participants from *n* = 43 at baseline to *n* = 20 at the end of the 12-month observation period. Estimated implementation according to the GEE model ranges from 98% (97–99%) at baseline to 97% (91–99%) at the end of the observation period. Persistence and adherence are represented in Figure 3. One can identify two specific time periods for discontinuation: the first 3 discontinuation events are responsible for the first decline happening within 60 days after study and OAM initiations; the last 2 discontinuation events are responsible for the second decline during the second semester of treatment. The probability of being persistent at 12 months was estimated at 85% (73% - 99%).

## Discussion

This study described a 12-month adherence -persistence and implementation- to different OAMs in a routine care medication adherence program, and characterized precisely reasons for discontinuation. While addressing these aims, this study had to focus on the methodological challenges of addressing adherence to OAMs. Indeed, the identification of the reasons for discontinuation to OAMs in real life clinical practice is challenging due to the high prevalence of permanent OAM stop not caused by non-persistence but due to various clinical decisions. Hence, this study describes persistence and implementation to six different OAMs by analyzing the reasons for treatment discontinuation. Two different time periods for non-persistence have been illustrated (one post-initiation and one in the second semester); they might suggest key critical phases of OAM medication adherence, highlighting the difficulty for OAM post-initiation as well as OAM toxicity development during the second semester of treatment. Further data and information are needed to consolidate these early findings. In persistent participants, the modeled implementation was high and remained stable over the 12-month observation period (from 98 to 97%), with an increase in variability.

Compared to other chronic diseases, treatment changes are more likely in oncology. Long-term OAMs involve numerous therapeutic changes due to treatment failure (e.g., resistance, mutations), alternated treatment plans (e.g., neoadjuvant treatments) or management of adverse events. Hence, the complex identification of non-persistence underlines the need to carefully and prospectively assess reasons for OAM stop or switch. Non-persistence could be misrepresented if for example interruption due to treatment completion or cancer relapse is not taken into account. This study defined non-persistence as any premature treatment discontinuation due to participant's own, unilateral decision and/or because of adverse effects. This definition is an attempt to put emphasis on adverse effects as the main reason for OAM discontinuation (Bassan et al., 2014; Kottschade and Lehner Reed, 2017).

The medication adherence *GEE* approach represents a statistical analysis that allowed us to describe implementation to OAM longitudinally over the study period. This modeling approach was used to characterize the participants' behavior with their treatment in routine outpatient care during a medication adherence program whereas literature mainly focuses on describing adherence in clinical trials within selected participant groups, not representing routine care (Gebbia et al., 2012; Jabbour et al., 2012; Krolop et al., 2013; Barthelemy et al., 2015; Lam and Cheung, 2016). Moreover, this methodological approach can be applied to analyze longitudinal, electronic monitoring of adherence to other drugs and in other diseases.

In the community, persistence in various chronic disease areas (e.g., diabetes, HIV, asthma) generally decreases with time but non-persistence is often tangled up with non-implementation.(Caro et al., 1999) In our study, persistence declined with time but implementation remained stable in persistent patients.

This study has some limitations. Firstly, the number of participants (*N* = 43) as well as the period of data collection (2008–2011) might limit the outreach of the study results. Nevertheless, the monitored OAMs are still in use with similar prescribing indications, arguing in favor of the applicability of the findings. Moreover, the questioning about the reasons for discontinuation is applicable to any current OAM. This question still remains relevant, as it has been poorly researched in cancer and chronic diseases so far and requires further investigation, for example whether they vary in naive or experimented patients. It would be important to verify these results in larger studies with new, targeted OAMs in various cancer types. The challenge to define non-persistence to OAMs in real practice is high, as a non-negligible part of the treatment interruptions are independent from a non-persistent behavior but could be falsely associated to non-persistence. Secondly, we decided to analyze all side effects and toxicity events as sources of non-persistence. This concept needs to be further addressed in the literature and a precise taxonomy for classifying reasons for discontinuation should be established as a reference guide.

This study was not powered enough to establish the associations between adherence to OAM and clinical outcomes. This will be the next experimental step with a larger group of participants, where the association between medication adherence, pharmacokinetics, pharmacodynamics, and patient outcomes will be investigated (Cardoso et al., 2018). Importantly, this study reveals that we should pay attention to the type of components—persistence or implementation- that we do measure and avoid mixing both. In routine care research, advanced statistical methods such as GEE are an interesting and alternate analysis to the establishment of thresholds for implementation, for example when such thresholds are impossible to establish as they may vary across molecules, cancer types, cancer stages, and may vary with time, for example, during the semester after initiation vs. long term OAM use.

## Conclusion

This study provides a first experience to estimate persistence and implementation electronically to various OAMs in four different cancer types. It points out the needs for both a better description of OAM adherence, defined as persistence and implementation, in real life practice and a better characterization of OAM discontinuation. Those are crucial elements of knowledge for understanding and strengthening research on OAM adherence. Our specific attempt to better define reasons for non-persistence offers new perspectives to increase the comparability of results across studies. Finally, this study underlines the need to transfer this knowledge into practice in order to support patient-tailored interventions in adherence, especially for newer OAM generations and in different types of cancers. Indeed, healthcare providers should help each individual patient achieve the highest personal level of adherence through educational patient-centered programs, balancing efficacy with patient safety, and quality of life. In the future, a better understanding of the relationship between adherence to OAM and drug blood levels is important to individualize the regimen and allow patients to get the best efficacy and safety outcomes (Cardoso et al., 2018).

## Author Contributions

MS was involved in following up participants at the medication adherence clinic, collected, and analyzed study data and wrote the manuscript. LJ was involved in writing the study protocol, screened and included participants, collected data, and reviewed the manuscript. BC screened and included participants, collected data, and reviewed the manuscript. CB wrote the manuscript. AW screened and included participants. OB and FL were involved in writing the study protocol. IL did the statistical analysis and wrote the manuscript.

### Conflict of Interest Statement

The authors declare that the research was conducted in the absence of any commercial or financial relationships that could be construed as a potential conflict of interest.

## References

[B1] Al-Barrak J. Cheung W. Y. (2013). Adherence to imatinib therapy in gastrointestinal stromal tumors and chronic myeloid leukemia. Supp. Care Cancer 21, 2351–2357. 10.1007/s00520-013-1831-6 23708821

[B2] Allen J. Williamson S. (2014). Over compliance with capecitabine oral chemotherapy. Int. J. Clin. Pharm. 36, 271–273. 10.1007/s11096-014-9921-1 24532364

[B3] Barillet M. Prevost V. Joly F. Clarisse B. (2015). Oral antineoplastic agents: how do we care about adherence? Br. J. Clin. Pharmacol. 80, 1289–1302. 10.1111/bcp.12734 26255807 PMC4693496

[B4] Barthelemy P. Asmane-De la Porte I. Meyer N. Duclos B. Serra S. Dourthe L-M.. (2015). Adherence and patients' attitudes to oral anticancer drugs: a prospective series of 201 patients focusing on targeted therapies. Oncology 88, 1–8. 10.1159/000366226 25247774

[B5] Bassan F. Peter F. Houbre B. Brennstuhl M. J. Costantini M. Speyer M. Tarquinio M.. (2014). Adherence to oral antineoplastic agents by cancer patients: definition and literature review. Eur. J. Cancer Care. 23, 22–35. 10.1111/ecc.12124 24079854

[B6] Bhattacharya D. Easthall C. Willoughby K. A. Small M. Watson S. (2012). Capecitabine non-adherence: exploration of magnitude, nature and contributing factors. J. Oncol. Pharm. Pract. 18, 333–342. 10.1177/1078155211436022 22298660

[B7] Blaschke T. F. Osterberg L. Vrijens B. Urquhart J. (2012). Adherence to medications: insights arising from studies on the unreliable link between prescribed and actual drug dosing histories. Annu Rev Pharmacol Toxicol. 52:275–301. 10.1146/annurev-pharmtox-011711-113247 21942628

[B8] Cardoso E. Csajka C. Schneider M. P. Widmer N. (2018). Effect of adherence on pharmacokinetic/pharmacodynamic relationships of oral targeted anticancer drugs. Clin Pharmacokinet. 57, 1–6. 10.1007/s40262-017-0571-z 28634655

[B9] Caro J. J. Salas M. Speckman J. L. Raggio G. Jackson J. D. (1999). Persistence with treatment for hypertension in actual practice. CMAJ. 160, 31–37. 9934341 PMC1229943

[B10] Cheung W. Y. Lai EC-C Ruan J. Y. Chang J. T. Setoguchi S. (2015). Comparative adherence to oral hormonal agents in older women with breast cancer. Breast Cancer Res. Treat. 152, 419–427. 10.1007/s10549-015-3455-7 26070268

[B11] Gebbia V. Bellavia G. Ferrau F. Valerio M. R. (2012). Adherence, compliance and persistence to oral antineoplastic therapy: a review focused on chemotherapeutic and biologic agents. Exp. Opin. Drug Safety. 11 (Suppl. 1), S49–59. 10.1517/14740338.2011.645803 22149481

[B12] Hershman D. L. Shao T. Kushi L. H. Buono D. Tsai W. Y. Fehrenbacher L.. (2011). Early discontinuation and non-adherence to adjuvant hormonal therapy are associated with increased mortality in women with breast cancer. Breast Cancer Res. Treat Breast Cancer Res. Treat. 126, 529–537. 10.1007/s10549-010-1132-4 20803066 PMC3462663

[B13] Ibrahim A. R. Eliasson L. Apperley J. F. Milojkovic D. Bua M. Szydlo R.. (2011). Poor adherence is the main reason for loss of ccyr and imatinib failure for chronic myeloid leukemia patients on long-term therapy. Blood 117, 3733–3736. 10.1182/blood-2010-10-309807 21346253 PMC6143152

[B14] Jabbour E. J. Kantarjian H. Eliasson L. Cornelison A. M. Marin D. (2012). Patient adherence to tyrosine kinase inhibitor therapy in chronic myeloid leukemia. Am. J. Hematol. 87, 687–691. 10.1002/ajh.23180 22473898 PMC11726351

[B15] Kottschade L. A. Lehner Reed M. (2017). Promoting oral therapy adherence: consensus statements from the faculty of the melanoma nursing initiative on oral melanoma therapies. Clin. J. Oncol. Nurs. 21(Suppl. 4):87–96. 10.1188/17.CJON.S4.87-96 28738053

[B16] Krolop L. Ko Y. D. Schwindt P. F. Schumacher C. Fimmers R. Jaehde U. (2013). Adherence management for patients with cancer taking capecitabine: a prospective two-arm cohort study. BMJ Open. 3:e003139. 10.1136/bmjopen-2013-003139 23872296 PMC3717446

[B17] Krummenacher I. Cavassini M. Bugnon O. Spirig R. Schneider M. P. (2010). Antiretroviral adherence program in HIV patients: a feasibility study in the Swiss HIV Cohort Study. Pharm. World Sci. 32, 776–786. 10.1007/s11096-010-9437-2 20862544

[B18] Lam M. S. Cheung N. (2016). Impact of oncology pharmacist-managed oral anticancer therapy in patients with chronic myelogenous leukemia. J. Oncol. Pharm. Pract. 22, 741–748. 10.1177/1078155215608523 26419691

[B19] Lehmann A. Aslani P. Ahmed R. Celio J. Gauchet A. Bedouch P.. (2014). Assessing medication adherence: options to consider. Int J Clin. Pharm. 36, 55–69. 10.1007/s11096-013-9865-x 24166659

[B20] Lelubre M. Kamal S. Genre N. Celio J. Gorgerat S. Hampai D. H.. (2015). Interdisciplinary medication adherence program: the example of a university community pharmacy in Switzerland. BioMed Res. Int. 2015:103546. 10.1155/2015/103546 26839879 PMC4709610

[B21] Nguyen T. M. U. Caze A. L. Cottrell N. (2014). What are validated self-report adherence scales really measuring?: a systematic review. Br. J. Clin. Pharmacol. 77, 427–445. 10.1111/bcp.12194 23803249 PMC3952718

[B22] Partridge A. H. Avorn J. Wang P. S. Winer E. P. (2002). Adherence to therapy with oral antineoplastic agents. J. Natl. Cancer Inst. 94, 652–661. 10.1093/jnci/94.9.652 11983753

[B23] Partridge A. H. Wang P. S. Winer E. P. Avorn J. (2003). Nonadherence to adjuvant tamoxifen therapy in women with primary breast cancer. J. Clin. Oncol. 21, 602–606. 10.1200/JCO.2003.07.071 12586795

[B24] R Core Team (2013). R: A Language and Environment for Statistical Computing. Vienna: R Foundation for Statistical Computing. Available online at: http://www.R-project.org/

[B25] Rotzinger A. Cavassini M. Bugnon O. Schneider M. P. (2016). Development of an algorithm for analysing the electronic measurement of medication adherence in routine HIV care. Int. J. Clin. Pharm. 38, 1210–1218. 10.1007/s11096-016-0354-x 27473709

[B26] Ruddy K. Mayer E. Partridge A. (2009). Patient adherence and persistence with oral anticancer treatment. CA Cancer J. Clin. 59, 56–66. 10.3322/caac.20004 19147869

[B27] Sabate E. (2003). Adherence to Long-Term Therapies: Evidence for Action. Geneve: World Health Organisation.

[B28] Timmers L. Boons C. C. Kropff F. van de Ven P. M. Swart E. L. Smit E. F.. (2014). Adherence and patients' experiences with the use of oral anticancer agents. Acta Oncol. 53, 259–267. 10.3109/0284186X.2013.844353 24266637

[B29] Verbrugghe M. Duprez V. Beeckman D. Grypdonck M. Quaghebeur M. Verschuere C.. (2016). Factors influencing adherence in cancer patients taking oral tyrosine kinase inhibitors: a qualitative Study. Cancer Nurs. 39, 153–162. 10.1097/NCC.0000000000000250 25815430

[B30] Vrijens B. Geest S. D. Hughes D. A. Przemyslaw K Demonceau J Ruppar T.. (2012). A new taxonomy for describing and defining adherence to medications. Br. J. Clin. Pharmacol. 73, 691–705. 10.1111/j.1365-2125.2012.04167.x 22486599 PMC3403197

[B31] Weingart S. N. Toro J. Spencer J. Duncombe D. Gross A. Bartel S.. (2010). Medication errors involving oral chemotherapy. Cancer. 116, 2455–2464. 10.1002/cncr.25027 20225328

[B32] Wood L. (2012). A review on adherence management in patients on oral cancer therapies. Eur. J Oncol. Nurs. 16, 432–438. 10.1016/j.ejon.2011.10.002 22051845

[B33] Zhang X. Zhang M.-J. Fine J. (2009). A mass redistribution algorithm for right censored and left truncated time to event data. J. Stat. Plan. Inference. 139, 3329–3339. 10.1016/j.jspi.2009.03.007 22553383 PMC3339805

